# {*N*,*N*-Dimethyl-*N*′-[1-(2-pyrid­yl)ethyl­idene]ethane-1,2-diamine-κ^3^ *N*,*N*′,*N*′′}bis­(thio­cyanato-κ*N*)copper(II)

**DOI:** 10.1107/S1600536811022057

**Published:** 2011-06-18

**Authors:** Nura Suleiman Gwaram, Hamid Khaledi, Hapipah Mohd Ali

**Affiliations:** a Department of Chemistry, University of Malaya, 50603 Kuala Lumpur, Malaysia

## Abstract

The asymmetric unit of the title compound, [Cu(NCS)_2_(C_11_H_17_N_3_)], consists of two crystallographically independent mol­ecules. In each mol­ecule, the Cu^II^ ion is five-coordinated in a distorted square-pyramidal geometry wherein the basal plane is defined by the *N,N′,N"*-tridentate Schiff base and one *N*-bound thio­cyanate ligand. The second *N*-donor thio­cyanate group, located at the apical site, completes the coordination environment. In the crystal, inter­molecular C—H⋯S and C—H⋯N hydrogen bonds link adjacent mol­ecules into infinite layers parallel to the *ac* plane. Intra­molecular C—H⋯N inter­actions are also observed.

## Related literature

For the structures of similar copper(II) isothio­cyanate complexes, see: Xue *et al.* (2010 ▶); Yue *et al.* (2005 ▶). For the structure of the polymeric cadmium thio­cyanate complex of the same Schiff base, see: Suleiman Gwaram *et al.* (2011 ▶). For a description of the geometry of complexes with five-coordinate metal atoms, see: Addison *et al.* (1984 ▶).
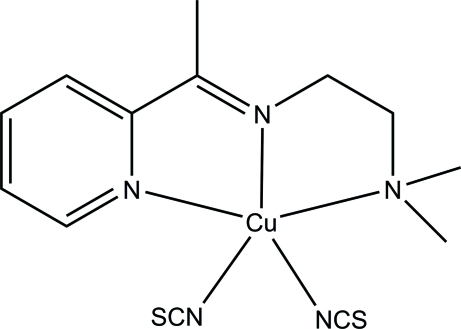


## Experimental

### 

#### Crystal data

[Cu(NCS)_2_(C_11_H_17_N_3_)]
*M* *_r_* = 370.98
Triclinic, 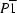

*a* = 10.9895 (4) Å
*b* = 11.2172 (4) Å
*c* = 13.7549 (5) Å
α = 81.222 (2)°
β = 87.121 (2)°
γ = 79.702 (2)°
*V* = 1648.27 (10) Å^3^
*Z* = 4
Mo *K*α radiation
μ = 1.58 mm^−1^
*T* = 100 K
0.44 × 0.31 × 0.13 mm

#### Data collection

Bruker APEXII CCD diffractometer
Absorption correction: multi-scan (*SADABS*; Sheldrick, 1996 ▶) *T*_min_ = 0.544, *T*_max_ = 0.821
14403 measured reflections
7159 independent reflections
5182 reflections with *I* > 2σ(*I*)
*R*_int_ = 0.036

#### Refinement

*R*[*F* ^2^ > 2σ(*F* ^2^)] = 0.045
*wR*(*F* ^2^) = 0.128
*S* = 1.00
7159 reflections
385 parameters
H-atom parameters constrained
Δρ_max_ = 1.04 e Å^−3^
Δρ_min_ = −0.81 e Å^−3^

### 

Data collection: *APEX2* (Bruker, 2007 ▶); cell refinement: *SAINT* (Bruker, 2007 ▶); data reduction: *SAINT*; program(s) used to solve structure: *SHELXS97* (Sheldrick, 2008 ▶); program(s) used to refine structure: *SHELXL97* (Sheldrick, 2008 ▶); molecular graphics: *X-SEED* (Barbour, 2001 ▶); software used to prepare material for publication: *SHELXL97* and *publCIF* (Westrip, 2010 ▶).

## Supplementary Material

Crystal structure: contains datablock(s) I, global. DOI: 10.1107/S1600536811022057/is2717sup1.cif

Structure factors: contains datablock(s) I. DOI: 10.1107/S1600536811022057/is2717Isup2.hkl

Additional supplementary materials:  crystallographic information; 3D view; checkCIF report

## Figures and Tables

**Table 1:** Hydrogen-bond geometry (Å, °)

*D*—H⋯*A*	*D*—H	H⋯*A*	*D*⋯*A*	*D*—H⋯*A*
C4—H4⋯N5^i^	0.95	2.58	3.429 (5)	149
C8—H8*B*⋯S4^ii^	0.99	2.82	3.788 (4)	167
C9—H9*A*⋯S4^iii^	0.99	2.84	3.735 (4)	150
C22—H22*A*⋯S2^iii^	0.99	2.81	3.749 (4)	159
C11—H11*B*⋯N5	0.98	2.60	3.156 (5)	116
C21—H21*A*⋯S1	0.99	2.84	3.727 (4)	150
C21—H21*B*⋯S2	0.99	2.82	3.793 (4)	167
C24—H24*B*⋯N10	0.98	2.60	3.193 (5)	119

Symmetry codes: (i) 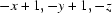; (ii) 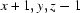; (iii) 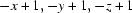.

**Fig. 1.**
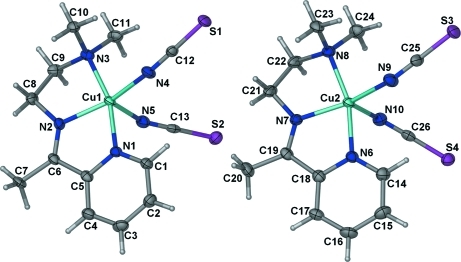
The molecular structure of the title compound (50% probability ellipsoids). Hydrogen atoms are drawn as spheres of arbitrary radius.

## supplementary crystallographic information

### Comment

The title compound was obtained upon the reaction of the Schiff base, *N,N*-dimethyl-*N'*-[methyl(2-pyridyl)methylene]ethane-1,2-diamine, with Cu^II^ ion in the presence of thiocyanate anion. There are two geometrically slightly different molecules in crystal structure. The weighted r.m.s. fit for the superposition of the non-H atoms in both molecules is 0.296 Å. Each metal ion is five-coordinated by the three N atoms from the Schiff base and two *N*-donor thiocyanate ligands. Similar arrangements are observed in the structures of related mixed-ligand copper(II) complexes (Xue *et al.*, 2010; Yue *et al.*, 2005). Different from those, in the cadmium(II) thiocyanate complex of the same Schiff base (Suleiman Gwaram *et al.*, 2011), *N:S* bridging thiocyanates connect the metal ions into an octahedral polymeric structure. The Addison τ values (Addison *et al.*, 1984) of 0.13 for Cu1 complex and 0.14 for Cu2 complex (τ = 0 for an ideal square pyramid and τ = 1 for an ideal trigonal bipyramid) imply distorted square-pyramidal geometries of the molecules. In the crystal, the adjacent molecules are bonded *via* C—H···S and C—H···N interactions (Table 1) into layers parallel to the *ac* plane. Moreover, intramolecular C—H···N hydrogen bonding occurs (Table 1).

### Experimental

A mixture of 2-acetylpyridine (0.2 g, 1.65 mmol) and *N,N*-dimethylethyldiamine (0.15 g, 1.65 mmol) in ethanol (20 ml) was refluxed. After 2 hr a solution of copper(II) chloride dihydrate (0.28 g, 1.65 mmol) and sodium thiocyanate (0.27 g, 3.3 mmol) in a minimum amount of water was added. The resulting solution was refluxed for an hour, then left at room temperature. The green crystals of the title compound were obtained in a few days.

### Refinement

The hydrogen atoms were placed at calculated positions and refined as riding atoms with C—H distances of 0.95 (aryl), 0.98 (methyl) and 0.99 (methylene) Å, and with*U*_iso_(H) set to 1.2(1.5 for methyl)*U*_eq_(C).

### Crystal data

**Table:**

[Cu(NCS)_2_(C_11_H_17_N_3_)]	*Z* = 4
*M**_r_* = 370.98	*F*(000) = 764
Triclinic, *P*1	*D*_x_ = 1.495 Mg m^−^^3^
Hall symbol: -P 1	Mo *K*α radiation, λ = 0.71073 Å
*a* = 10.9895 (4) Å	Cell parameters from 3803 reflections
*b* = 11.2172 (4) Å	θ = 2.4–27.5°
*c* = 13.7549 (5) Å	µ = 1.58 mm^−^^1^
α = 81.222 (2)°	*T* = 100 K
β = 87.121 (2)°	Block, green
γ = 79.702 (2)°	0.44 × 0.31 × 0.13 mm
*V* = 1648.27 (10) Å^3^	

### Data collection

**Table:**

Bruker APEXII CCD diffractometer	7159 independent reflections
Radiation source: fine-focus sealed tube	5182 reflections with *I* > 2σ(*I*)
graphite	*R*_int_ = 0.036
φ and ω scans	θ_max_ = 27.0°, θ_min_ = 1.9°
Absorption correction: multi-scan (*SADABS*; Sheldrick, 1996)	*h* = −14→14
*T*_min_ = 0.544, *T*_max_ = 0.821	*k* = −14→14
14403 measured reflections	*l* = −17→17

### Refinement

**Table:**

Refinement on *F*^2^	Primary atom site location: structure-invariant direct methods
Least-squares matrix: full	Secondary atom site location: difference Fourier map
*R*[*F*^2^ > 2σ(*F*^2^)] = 0.045	Hydrogen site location: inferred from neighbouring sites
*wR*(*F*^2^) = 0.128	H-atom parameters constrained
*S* = 1.00	*w* = 1/[σ^2^(*F*_o_^2^) + (0.0704*P*)^2^ + 0.4086*P*] where *P* = (*F*_o_^2^ + 2*F*_c_^2^)/3
7159 reflections	(Δ/σ)_max_ = 0.001
385 parameters	Δρ_max_ = 1.04 e Å^−^^3^
0 restraints	Δρ_min_ = −0.81 e Å^−^^3^

### Special details

**Table:**

Geometry. All e.s.d.'s (except the e.s.d. in the dihedral angle between two l.s. planes) are estimated using the full covariance matrix. The cell e.s.d.'s are taken into account individually in the estimation of e.s.d.'s in distances, angles and torsion angles; correlations between e.s.d.'s in cell parameters are only used when they are defined by crystal symmetry. An approximate (isotropic) treatment of cell e.s.d.'s is used for estimating e.s.d.'s involving l.s. planes.
Refinement. Refinement of *F*^2^ against ALL reflections. The weighted *R*-factor *wR* and goodness of fit *S* are based on *F*^2^, conventional *R*-factors *R* are based on *F*, with *F* set to zero for negative *F*^2^. The threshold expression of *F*^2^ > σ(*F*^2^) is used only for calculating *R*-factors(gt) *etc*. and is not relevant to the choice of reflections for refinement. *R*-factors based on *F*^2^ are statistically about twice as large as those based on *F*, and *R*- factors based on ALL data will be even larger.

### Fractional atomic coordinates and isotropic or equivalent isotropic displacement parameters (Å^2^)

**Table:**

	*x*	*y*	*z*	*U*_iso_*/*U*_eq_	
Cu1	0.55642 (4)	0.79503 (4)	0.08656 (3)	0.02048 (12)	
S1	0.38800 (9)	1.07075 (9)	0.30413 (7)	0.0312 (2)	
S2	0.51073 (9)	0.51728 (9)	0.37652 (7)	0.0290 (2)	
N1	0.4089 (3)	0.7396 (2)	0.0358 (2)	0.0205 (6)	
N2	0.6288 (3)	0.7362 (3)	−0.0344 (2)	0.0222 (6)	
N3	0.7243 (3)	0.8449 (3)	0.0977 (2)	0.0226 (6)	
N4	0.4644 (3)	0.9151 (3)	0.1652 (2)	0.0281 (7)	
N5	0.5861 (3)	0.6353 (3)	0.1947 (2)	0.0290 (7)	
C1	0.2960 (3)	0.7478 (3)	0.0763 (3)	0.0256 (8)	
H1	0.2764	0.7927	0.1300	0.031*	
C2	0.2057 (4)	0.6931 (3)	0.0429 (3)	0.0300 (9)	
H2	0.1252	0.7011	0.0726	0.036*	
C3	0.2350 (4)	0.6270 (3)	−0.0341 (3)	0.0313 (9)	
H3	0.1750	0.5879	−0.0578	0.038*	
C4	0.3526 (4)	0.6180 (3)	−0.0771 (3)	0.0273 (8)	
H4	0.3743	0.5727	−0.1302	0.033*	
C5	0.4380 (3)	0.6765 (3)	−0.0408 (2)	0.0215 (7)	
C6	0.5665 (3)	0.6775 (3)	−0.0811 (2)	0.0227 (8)	
C7	0.6097 (4)	0.6144 (3)	−0.1675 (3)	0.0291 (9)	
H7A	0.5676	0.6601	−0.2264	0.044*	
H7B	0.5907	0.5313	−0.1559	0.044*	
H7C	0.6992	0.6105	−0.1770	0.044*	
C8	0.7556 (3)	0.7571 (3)	−0.0580 (3)	0.0264 (8)	
H8A	0.7552	0.8340	−0.1043	0.032*	
H8B	0.8037	0.6885	−0.0886	0.032*	
C9	0.8114 (3)	0.7658 (3)	0.0381 (3)	0.0277 (8)	
H9A	0.8328	0.6828	0.0759	0.033*	
H9B	0.8886	0.8000	0.0246	0.033*	
C10	0.7179 (3)	0.9758 (3)	0.0576 (3)	0.0252 (8)	
H10A	0.6568	1.0257	0.0958	0.038*	
H10B	0.6936	0.9896	−0.0113	0.038*	
H10C	0.7992	0.9988	0.0618	0.038*	
C11	0.7663 (4)	0.8237 (3)	0.2004 (3)	0.0298 (9)	
H11A	0.8478	0.8474	0.2019	0.045*	
H11B	0.7721	0.7367	0.2269	0.045*	
H11C	0.7070	0.8729	0.2404	0.045*	
C12	0.4320 (3)	0.9790 (3)	0.2235 (3)	0.0229 (7)	
C13	0.5539 (3)	0.5866 (3)	0.2700 (3)	0.0230 (8)	
Cu2	0.08751 (4)	0.80111 (4)	0.59197 (3)	0.02222 (13)	
S3	−0.06785 (10)	1.01400 (10)	0.84292 (8)	0.0375 (3)	
S4	−0.01284 (9)	0.51398 (8)	0.83703 (7)	0.0290 (2)	
N6	−0.0442 (3)	0.7242 (3)	0.5384 (2)	0.0230 (6)	
N7	0.1704 (3)	0.7564 (3)	0.4703 (2)	0.0253 (7)	
N8	0.2403 (3)	0.8784 (3)	0.6043 (2)	0.0253 (7)	
N9	−0.0218 (3)	0.9089 (3)	0.6712 (2)	0.0321 (8)	
N10	0.1343 (3)	0.6425 (3)	0.7029 (2)	0.0320 (8)	
C14	−0.1518 (3)	0.7069 (3)	0.5813 (3)	0.0289 (8)	
H14	−0.1759	0.7387	0.6409	0.035*	
C15	−0.2304 (4)	0.6438 (3)	0.5414 (3)	0.0332 (9)	
H15	−0.3064	0.6312	0.5738	0.040*	
C16	−0.1958 (4)	0.5994 (3)	0.4535 (3)	0.0327 (9)	
H16	−0.2486	0.5573	0.4241	0.039*	
C17	−0.0834 (4)	0.6172 (3)	0.4092 (3)	0.0275 (8)	
H17	−0.0578	0.5869	0.3493	0.033*	
C18	−0.0083 (3)	0.6801 (3)	0.4532 (3)	0.0244 (8)	
C19	0.1145 (3)	0.7029 (3)	0.4143 (3)	0.0237 (8)	
C20	0.1644 (4)	0.6699 (4)	0.3174 (3)	0.0353 (9)	
H20A	0.1340	0.7366	0.2650	0.053*	
H20B	0.1370	0.5947	0.3061	0.053*	
H20C	0.2550	0.6567	0.3172	0.053*	
C21	0.2912 (4)	0.7928 (4)	0.4488 (3)	0.0332 (9)	
H21A	0.2832	0.8705	0.4026	0.040*	
H21B	0.3483	0.7288	0.4189	0.040*	
C22	0.3390 (4)	0.8088 (4)	0.5468 (3)	0.0322 (9)	
H22A	0.3685	0.7274	0.5850	0.039*	
H22B	0.4099	0.8531	0.5351	0.039*	
C23	0.2171 (4)	1.0098 (3)	0.5624 (3)	0.0301 (9)	
H23A	0.1507	1.0536	0.6005	0.045*	
H23B	0.1926	1.0188	0.4938	0.045*	
H23C	0.2927	1.0440	0.5652	0.045*	
C24	0.2786 (4)	0.8646 (4)	0.7075 (3)	0.0330 (9)	
H24A	0.3551	0.8975	0.7097	0.049*	
H24B	0.2928	0.7776	0.7355	0.049*	
H24C	0.2133	0.9095	0.7459	0.049*	
C25	−0.0419 (3)	0.9524 (3)	0.7423 (3)	0.0269 (8)	
C26	0.0736 (3)	0.5899 (3)	0.7593 (3)	0.0247 (8)	

### Atomic displacement parameters (Å^2^)

**Table:**

	*U*^11^	*U*^22^	*U*^33^	*U*^12^	*U*^13^	*U*^23^
Cu1	0.0231 (2)	0.0194 (2)	0.0209 (2)	−0.00395 (17)	−0.00033 (17)	−0.00881 (17)
S1	0.0327 (6)	0.0299 (5)	0.0359 (5)	−0.0084 (4)	0.0083 (4)	−0.0195 (4)
S2	0.0289 (5)	0.0300 (5)	0.0285 (5)	−0.0090 (4)	−0.0006 (4)	−0.0010 (4)
N1	0.0239 (16)	0.0159 (14)	0.0224 (15)	−0.0039 (12)	−0.0005 (12)	−0.0040 (11)
N2	0.0239 (16)	0.0212 (15)	0.0217 (15)	−0.0031 (12)	0.0031 (12)	−0.0061 (12)
N3	0.0253 (17)	0.0181 (14)	0.0245 (15)	−0.0017 (12)	−0.0061 (13)	−0.0039 (12)
N4	0.0280 (18)	0.0284 (16)	0.0307 (17)	−0.0039 (13)	−0.0008 (14)	−0.0143 (14)
N5	0.038 (2)	0.0242 (16)	0.0263 (17)	−0.0086 (14)	−0.0016 (14)	−0.0047 (14)
C1	0.028 (2)	0.0251 (18)	0.0262 (19)	−0.0065 (15)	0.0023 (16)	−0.0093 (15)
C2	0.026 (2)	0.032 (2)	0.033 (2)	−0.0090 (16)	0.0027 (17)	−0.0048 (17)
C3	0.031 (2)	0.033 (2)	0.033 (2)	−0.0126 (17)	−0.0093 (17)	−0.0023 (17)
C4	0.035 (2)	0.0240 (18)	0.0250 (19)	−0.0076 (16)	−0.0027 (16)	−0.0057 (15)
C5	0.027 (2)	0.0144 (16)	0.0219 (17)	−0.0003 (14)	−0.0017 (15)	−0.0033 (13)
C6	0.030 (2)	0.0175 (16)	0.0209 (17)	−0.0021 (14)	0.0004 (15)	−0.0060 (14)
C7	0.038 (2)	0.0267 (19)	0.0265 (19)	−0.0081 (17)	0.0019 (17)	−0.0148 (16)
C8	0.023 (2)	0.0262 (19)	0.031 (2)	−0.0033 (15)	0.0059 (16)	−0.0122 (16)
C9	0.021 (2)	0.0233 (18)	0.039 (2)	−0.0006 (15)	−0.0036 (16)	−0.0082 (16)
C10	0.030 (2)	0.0203 (17)	0.0271 (19)	−0.0082 (15)	0.0000 (16)	−0.0041 (15)
C11	0.034 (2)	0.030 (2)	0.027 (2)	−0.0109 (17)	−0.0107 (17)	−0.0016 (16)
C12	0.0179 (18)	0.0253 (18)	0.0273 (19)	−0.0066 (14)	0.0010 (15)	−0.0062 (15)
C13	0.0209 (19)	0.0193 (17)	0.032 (2)	−0.0038 (14)	−0.0049 (16)	−0.0121 (15)
Cu2	0.0214 (2)	0.0216 (2)	0.0258 (2)	−0.00454 (17)	0.00123 (18)	−0.01010 (18)
S3	0.0373 (6)	0.0411 (6)	0.0403 (6)	−0.0126 (5)	0.0130 (5)	−0.0238 (5)
S4	0.0276 (5)	0.0255 (5)	0.0349 (5)	−0.0060 (4)	0.0034 (4)	−0.0071 (4)
N6	0.0242 (17)	0.0197 (15)	0.0254 (15)	−0.0028 (12)	−0.0001 (13)	−0.0057 (12)
N7	0.0256 (17)	0.0216 (15)	0.0307 (17)	−0.0072 (12)	0.0040 (13)	−0.0086 (13)
N8	0.0261 (17)	0.0199 (15)	0.0310 (17)	−0.0048 (12)	0.0012 (13)	−0.0070 (13)
N9	0.0260 (19)	0.0334 (18)	0.0396 (19)	−0.0033 (14)	0.0020 (15)	−0.0169 (16)
N10	0.038 (2)	0.0290 (17)	0.0307 (17)	−0.0113 (15)	0.0009 (15)	−0.0046 (14)
C14	0.023 (2)	0.029 (2)	0.035 (2)	−0.0029 (15)	0.0008 (16)	−0.0079 (16)
C15	0.023 (2)	0.030 (2)	0.048 (2)	−0.0080 (16)	−0.0035 (18)	−0.0027 (18)
C16	0.032 (2)	0.029 (2)	0.040 (2)	−0.0062 (17)	−0.0134 (18)	−0.0096 (18)
C17	0.031 (2)	0.0226 (18)	0.0296 (19)	−0.0025 (15)	−0.0069 (16)	−0.0080 (15)
C18	0.028 (2)	0.0191 (17)	0.0248 (18)	−0.0014 (15)	−0.0027 (15)	−0.0015 (14)
C19	0.025 (2)	0.0192 (17)	0.0254 (18)	0.0023 (14)	−0.0012 (15)	−0.0068 (14)
C20	0.044 (3)	0.035 (2)	0.029 (2)	−0.0064 (18)	0.0054 (18)	−0.0130 (18)
C21	0.030 (2)	0.028 (2)	0.046 (2)	−0.0113 (17)	0.0136 (19)	−0.0180 (18)
C22	0.022 (2)	0.029 (2)	0.049 (2)	−0.0079 (16)	0.0079 (18)	−0.0161 (18)
C23	0.039 (2)	0.0213 (18)	0.031 (2)	−0.0071 (16)	0.0014 (17)	−0.0085 (16)
C24	0.032 (2)	0.035 (2)	0.032 (2)	−0.0082 (17)	−0.0069 (17)	−0.0029 (18)
C25	0.0207 (19)	0.0242 (18)	0.038 (2)	−0.0046 (15)	0.0060 (16)	−0.0126 (17)
C26	0.028 (2)	0.0231 (18)	0.0246 (19)	−0.0012 (15)	−0.0080 (16)	−0.0095 (15)

### Geometric parameters (Å, °)

**Table:**

Cu1—N2	1.963 (3)	Cu2—N7	1.955 (3)
Cu1—N4	1.963 (3)	Cu2—N9	1.961 (3)
Cu1—N1	2.027 (3)	Cu2—N6	2.033 (3)
Cu1—N3	2.040 (3)	Cu2—N8	2.048 (3)
Cu1—N5	2.134 (3)	Cu2—N10	2.164 (3)
S1—C12	1.625 (4)	S3—C25	1.630 (4)
S2—C13	1.640 (4)	S4—C26	1.637 (4)
N1—C1	1.328 (5)	N6—C14	1.327 (5)
N1—C5	1.351 (4)	N6—C18	1.354 (4)
N2—C6	1.284 (4)	N7—C19	1.284 (5)
N2—C8	1.466 (4)	N7—C21	1.461 (5)
N3—C10	1.478 (4)	N8—C24	1.479 (5)
N3—C11	1.480 (4)	N8—C23	1.480 (4)
N3—C9	1.489 (5)	N8—C22	1.487 (5)
N4—C12	1.157 (4)	N9—C25	1.153 (5)
N5—C13	1.165 (5)	N10—C26	1.159 (5)
C1—C2	1.386 (5)	C14—C15	1.390 (5)
C1—H1	0.9500	C14—H14	0.9500
C2—C3	1.377 (5)	C15—C16	1.387 (6)
C2—H2	0.9500	C15—H15	0.9500
C3—C4	1.387 (5)	C16—C17	1.383 (5)
C3—H3	0.9500	C16—H16	0.9500
C4—C5	1.388 (5)	C17—C18	1.391 (5)
C4—H4	0.9500	C17—H17	0.9500
C5—C6	1.493 (5)	C18—C19	1.477 (5)
C6—C7	1.489 (5)	C19—C20	1.490 (5)
C7—H7A	0.9800	C20—H20A	0.9800
C7—H7B	0.9800	C20—H20B	0.9800
C7—H7C	0.9800	C20—H20C	0.9800
C8—C9	1.508 (5)	C21—C22	1.518 (6)
C8—H8A	0.9900	C21—H21A	0.9900
C8—H8B	0.9900	C21—H21B	0.9900
C9—H9A	0.9900	C22—H22A	0.9900
C9—H9B	0.9900	C22—H22B	0.9900
C10—H10A	0.9800	C23—H23A	0.9800
C10—H10B	0.9800	C23—H23B	0.9800
C10—H10C	0.9800	C23—H23C	0.9800
C11—H11A	0.9800	C24—H24A	0.9800
C11—H11B	0.9800	C24—H24B	0.9800
C11—H11C	0.9800	C24—H24C	0.9800
			
N2—Cu1—N4	155.48 (13)	N7—Cu2—N9	154.58 (14)
N2—Cu1—N1	79.56 (12)	N7—Cu2—N6	79.81 (12)
N4—Cu1—N1	97.64 (12)	N9—Cu2—N6	97.93 (13)
N2—Cu1—N3	84.28 (12)	N7—Cu2—N8	83.79 (12)
N4—Cu1—N3	95.54 (12)	N9—Cu2—N8	95.01 (13)
N1—Cu1—N3	163.43 (12)	N6—Cu2—N8	163.03 (12)
N2—Cu1—N5	103.65 (12)	N7—Cu2—N10	105.98 (12)
N4—Cu1—N5	100.73 (13)	N9—Cu2—N10	99.36 (13)
N1—Cu1—N5	90.70 (12)	N6—Cu2—N10	91.09 (12)
N3—Cu1—N5	96.68 (12)	N8—Cu2—N10	97.61 (12)
C1—N1—C5	119.6 (3)	C14—N6—C18	120.1 (3)
C1—N1—Cu1	126.8 (2)	C14—N6—Cu2	126.9 (3)
C5—N1—Cu1	113.1 (2)	C18—N6—Cu2	112.8 (2)
C6—N2—C8	126.4 (3)	C19—N7—C21	125.3 (3)
C6—N2—Cu1	118.7 (2)	C19—N7—Cu2	119.0 (3)
C8—N2—Cu1	114.8 (2)	C21—N7—Cu2	115.7 (2)
C10—N3—C11	109.2 (3)	C24—N8—C23	109.6 (3)
C10—N3—C9	110.9 (3)	C24—N8—C22	109.2 (3)
C11—N3—C9	109.4 (3)	C23—N8—C22	110.8 (3)
C10—N3—Cu1	109.8 (2)	C24—N8—Cu2	112.1 (2)
C11—N3—Cu1	112.5 (2)	C23—N8—Cu2	110.6 (2)
C9—N3—Cu1	105.0 (2)	C22—N8—Cu2	104.5 (2)
C12—N4—Cu1	165.6 (3)	C25—N9—Cu2	150.1 (3)
C13—N5—Cu1	146.3 (3)	C26—N10—Cu2	131.7 (3)
N1—C1—C2	122.2 (3)	N6—C14—C15	121.8 (4)
N1—C1—H1	118.9	N6—C14—H14	119.1
C2—C1—H1	118.9	C15—C14—H14	119.1
C3—C2—C1	118.6 (4)	C16—C15—C14	118.9 (4)
C3—C2—H2	120.7	C16—C15—H15	120.6
C1—C2—H2	120.7	C14—C15—H15	120.6
C2—C3—C4	119.6 (3)	C17—C16—C15	119.1 (3)
C2—C3—H3	120.2	C17—C16—H16	120.5
C4—C3—H3	120.2	C15—C16—H16	120.5
C3—C4—C5	118.7 (3)	C16—C17—C18	119.3 (4)
C3—C4—H4	120.7	C16—C17—H17	120.3
C5—C4—H4	120.7	C18—C17—H17	120.3
N1—C5—C4	121.2 (3)	N6—C18—C17	120.8 (3)
N1—C5—C6	114.1 (3)	N6—C18—C19	114.7 (3)
C4—C5—C6	124.6 (3)	C17—C18—C19	124.5 (3)
N2—C6—C7	126.9 (3)	N7—C19—C18	113.6 (3)
N2—C6—C5	113.3 (3)	N7—C19—C20	124.3 (3)
C7—C6—C5	119.8 (3)	C18—C19—C20	122.0 (3)
C6—C7—H7A	109.5	C19—C20—H20A	109.5
C6—C7—H7B	109.5	C19—C20—H20B	109.5
H7A—C7—H7B	109.5	H20A—C20—H20B	109.5
C6—C7—H7C	109.5	C19—C20—H20C	109.5
H7A—C7—H7C	109.5	H20A—C20—H20C	109.5
H7B—C7—H7C	109.5	H20B—C20—H20C	109.5
N2—C8—C9	106.4 (3)	N7—C21—C22	106.0 (3)
N2—C8—H8A	110.4	N7—C21—H21A	110.5
C9—C8—H8A	110.4	C22—C21—H21A	110.5
N2—C8—H8B	110.4	N7—C21—H21B	110.5
C9—C8—H8B	110.4	C22—C21—H21B	110.5
H8A—C8—H8B	108.6	H21A—C21—H21B	108.7
N3—C9—C8	111.3 (3)	N8—C22—C21	110.9 (3)
N3—C9—H9A	109.4	N8—C22—H22A	109.5
C8—C9—H9A	109.4	C21—C22—H22A	109.5
N3—C9—H9B	109.4	N8—C22—H22B	109.5
C8—C9—H9B	109.4	C21—C22—H22B	109.5
H9A—C9—H9B	108.0	H22A—C22—H22B	108.1
N3—C10—H10A	109.5	N8—C23—H23A	109.5
N3—C10—H10B	109.5	N8—C23—H23B	109.5
H10A—C10—H10B	109.5	H23A—C23—H23B	109.5
N3—C10—H10C	109.5	N8—C23—H23C	109.5
H10A—C10—H10C	109.5	H23A—C23—H23C	109.5
H10B—C10—H10C	109.5	H23B—C23—H23C	109.5
N3—C11—H11A	109.5	N8—C24—H24A	109.5
N3—C11—H11B	109.5	N8—C24—H24B	109.5
H11A—C11—H11B	109.5	H24A—C24—H24B	109.5
N3—C11—H11C	109.5	N8—C24—H24C	109.5
H11A—C11—H11C	109.5	H24A—C24—H24C	109.5
H11B—C11—H11C	109.5	H24B—C24—H24C	109.5
N4—C12—S1	178.8 (3)	N9—C25—S3	179.1 (4)
N5—C13—S2	179.1 (4)	N10—C26—S4	178.7 (3)

### Hydrogen-bond geometry (Å, °)

**Table:**

*D*—H···*A*	*D*—H	H···*A*	*D*···*A*	*D*—H···*A*
C4—H4···N5^i^	0.95	2.58	3.429 (5)	149
C8—H8B···S4^ii^	0.99	2.82	3.788 (4)	167
C9—H9A···S4^iii^	0.99	2.84	3.735 (4)	150
C22—H22A···S2^iii^	0.99	2.81	3.749 (4)	159
C11—H11B···N5	0.98	2.60	3.156 (5)	116
C21—H21A···S1	0.99	2.84	3.727 (4)	150
C21—H21B···S2	0.99	2.82	3.793 (4)	167
C24—H24B···N10	0.98	2.60	3.193 (5)	119

Symmetry codes: (i) −*x*+1, −*y*+1, −*z*; (ii) *x*+1, *y*, *z*−1; (iii) −*x*+1, −*y*+1, −*z*+1.

## References

[bb1] Addison, A. W., Rao, T. N., Reedijk, J., Rijn, V. J. & Verschoor, G. C. (1984). *J. Chem. Soc. Dalton Trans.* pp. 1349–1356.

[bb2] Barbour, L. J. (2001). *J. Supramol. Chem* **1**, 189–191.

[bb3] Bruker (2007). *APEX2* and *SAINT* Bruker AXS Inc., Madison, Wisconsin, USA.

[bb4] Sheldrick, G. M. (1996). University of Göttingen, Germany.

[bb5] Sheldrick, G. M. (2008). *Acta Cryst.* A**64**, 112–122. 10.1107/S0108767307043930 18156677

[bb6] Suleiman Gwaram, N., Khaledi, H. & Mohd Ali, H. (2011). *Acta Cryst.* E**67**, m480. 10.1107/S1600536811010063 PMC3099776 21753992

[bb7] Westrip, S. P. (2010). *J. Appl. Cryst.* **43**, 920–925.

[bb8] Xue, L.-W., Zhao, G.-Q., Han, Y.-J., Chen, L.-H. & Peng, Q.-L. (2010). *Acta Cryst.* E**66**, m1274. 10.1107/S1600536810036378 PMC2983150 21587418

[bb9] Yue, G.-R., Xu, X.-J., Shi, Y.-Z. & Feng, L. (2005). *Acta Cryst.* E**61**, m693–m694.

